# Hydrothermal Carbonization and Pellet Production from *Egeria densa* and *Lemna minor*

**DOI:** 10.3390/plants9040425

**Published:** 2020-03-31

**Authors:** Xana Álvarez, Ángeles Cancela, Vanesa Freitas, Enrique Valero, Ángel Sánchez, Carolina Acuña-Alonso

**Affiliations:** 1 Department of Natural Resources Engineering and the Environment, School of Forestry Engineering, University of Vigo, Campus A Xunqueira s/n., 36005 Vigo, Pontevedra, Spain; evalero@uvigo.es (E.V.); carolina.alonso@uvigo.es (C.A.-A.); 2 Chemical Engineering Department, School of Forestry Engineering, Campus A Xunqueira s/n, University of Vigo, 36005 Vigo, Pontevedra, Spain; chiqui@uvigo.es; 3 School of Forestry Engineering, University of Vigo, Campus A Xunqueira s/n., 36005 Vigo, Pontevedra, Spain; vanesa.fb.94@gmail.com; 4 Chemical Engineering Department, Industrial Engineering College, University of Vigo, Campus Lagoas-Marcosende s/n, 36310 Vigo, Pontevedra, Spain; asanchez@uvigo.es

**Keywords:** Aquatic plants, *Egeria densa*, *Lemna minor*, hydrothermal carbonization, pellets

## Abstract

Biofuels are seen as a potential option for mitigating the effects of fossil fuel use. On the other hand, nutrient pollution is accelerating eutrophication rates in rivers, lakes, and coastal waters. Harvesting aquatic plants to produce biofuels could mitigate this problem, though it is important to attack the problem at source, mainly as regards the contribution of nutrients. For the first time, solid biofuels were obtained in the forms of carbon and pellets from the aquatic plants *Egeria densa*, which is classed as an invasive plant under the Spanish Catalogue of Exotic Invasive Species, and *Lemna minor*, both of which can be found in the Umia River in north-west Spain. The essential oils and macro- and microelements present in both these plants were also extracted and analyzed. The higher heating values (HHVs) of the carbon products obtained ranged from 14.28 to 17.25 MJ/kg. The ash content ranged from 22.69% to 49.57%. The maximum yield obtained for biochar for *Egeria densa* at 200 °C was 66.89%. Temperature significantly affects solid hydrochar yield. The HHVs of the pellets obtained ranged from 11.38 to 13.49 MJ/kg. The use of these species to obtain biofuels through hydrothermal carbonization (HTC) and pellets is a novel and effective approach that will facilitate the removal of nutrients that cause eutrophication in the Umia River. The elements extracted show that harvesting these plants will help to remove excessive nutrients from the ecosystem.

## 1. Introduction

Biofuels are seen as a potential option for mitigating the effects of fossil fuel use and thus reducing greenhouse gas (GHG) emissions [1,2]. First and second-generation biofuels mainly use food crops, such as corn, sugar beet, and sugarcane, and plant waste biomass, such as agriculture and forest residue [3], plus short rotation plantations of willows, poplars, miscanthus [4], and paulownia [5]. However, this has some limitations: Some of the resulting biofuels require vast amounts of arable land and compete directly with food crop use [6,7]. Using food as a fuel source has also increased the price of traditional foods [6,8].

On the other hand, nutrient pollution is accelerating eutrophication rates in water in numerous areas [9]. Eutrophication is a phenomenon associated with an excess of macro nutrients, especially nitrogen and phosphorus, caused mainly by human activities [10]. This excess of nutrients can lead to excess plant growth (and therefore to the exclusion of less competitive species [11]) and to fish death [12]. Using aquatic plants as biomass to produce biofuels could solve this problem. As a source of biofuels, aquatic plants have the benefit of not competing with grains and vegetables on arable land [13]. Aquatic plants use pollutants, such as nitrogen and phosphorus, as nutrients, removing them from water [9]. They do not add CO_2_ or heat to the atmosphere but actually recycle them [8]. Aquatic plants can colonize wetlands, grow in wastewater, and produce large amounts of biomass [14,15]. Mishima et al. [16] evaluated the viability of water lettuce (*Pistia stratiotes*) and water hyacinth (*Eichhornia crassipes*) as sources for ethanol. Their results showed that the ethanol yields from both species are similar to those from other agricultural wastes, and could therefore be used for ethanol production. *Typha* (cattail) species, for example, are characterized by their high productivity, pest resistance, and adaptability [17]. They also contain about 47.6% cellulose and 22% lignin, making cattail a good source of biofuel [18].

The main objective of the present study was to obtain, for the first time, solid biofuels in the form of carbon and pellets from the aquatic plants *Egeria densa*, which is considered an invasive plant under the Spanish Catalogue of Exotic Invasive Species, and *Lemna minor.* These plants do not compete with agriculture or forestry for land and fresh water [19]. These two plants grow along the Umia River in north-west Spain, where they are increasing in abundance, leading to a reduction in light and oxygen for other species and increasing the eutrophication of the ecosystem. Seeking to solve an environmental problem, this study analysed the feasibility of removing these plants from the aquatic environment and obtaining energy from them. This would reduce their environmental impact and the process would be optimized by obtaining energy from them. This was not done for commercial purposes but with the aim of reducing the amount of waste produced. The intention was to study how a carbon-rich solid product named hydrochar [20] can be obtained via a process named hydrothermal carbonization (HTC). This process also results in bio-gas and bio-oil [21]. The advantage of HTC is that biomass can be converted into carbonaceous solids with no need for an energy-intensive drying process before or during the HTC process [22]. HTC applied to the production of solid fuel with algal biomass is considered a renewable energy [23]. The innovative aspect of this study is that the potential application of this process to plants that cause eutrophication in fresh water and its possible use in boilers in pellet form were analyzed. As a secondary objective, extracts from these plants were also obtained. Different ways of using these plants were thus analyzed, with a view to reducing their impact on the aquatic ecosystem.

## 2. Results and Discussion

### 2.1. Extraction of Essential Oils, Macro- and Microelements, and Protein

The extraction yield of essential oils obtained from *L. minor* was 3.5 ± 0.2% of the sample’s initial weight. The extraction yields of essential oils obtained from *E. densa* were 13.3 ± 1.12% for ethanol, 16 ± 0.32% for acetone, and 19.6 ± 23.5% for water. A higher performance was obtained when the extraction was performed with water, which is also the most economical method. This finding contrasts with other studies [24]. These results are high compared to those of other authors. Li et al. [25] extracted essential oils from sea buckthorn (*Hippophae rhamnoides*) leaves using ionic liquid-based ultrasound/microwave-assisted simultaneous distillation extraction (ILUMASDE) and obtained an extraction yield of 0.095%. Zhi-ling et al. [26], obtained an extraction yield of 4.80 % from lavender using supercritical carbon dioxide extraction (SC-CO2). The same method was applied by Wu et al. [27] to obtain essential oils from citronella (*Cymbopogon citronella*), with an extraction yield of 4.4%. It is, however, important to take into account what solvents and amounts were used, as these factors can give different extraction yields. Using a soxhlet extractor is not only a novel approach to obtaining essential oils from these plants but also a simple and effective method. These comparisons are with species different from those studied here. The present study is also innovative in the sense that there are no previous analyses of the same plants.

The nitrogen (N) and carbon (C) concentrations are shown in Table 1. In both cases, the figures are very similar for the two species. Macroelements and microelements, such as potassium (K), copper (Cu), nickel (Ni), chromium (Cr), cadmium (Cd), lead (Pb), and selenium (Se), were found in higher amounts in *E. densa* than in *L. minor* (Table 1).

The protein content is similar in both species. *Lemna minor* has the highest content of protein with 24.81 ± 1.63%, while that of *E. densa* is 22.90 ± 1.36%. Several authors have studied the use of aquatic plants as supplementary food, mainly due to their availability and affordability [28]. Dewanji et al. [29] analyzed the protein content of 30 aquatic plants, including *L. minor*. Their results showed a protein nitrogen extractability content for *L. minor* of 21.49%. Bahnasy et al. [30] studied the use of dried aquatic plants as a protein source for animals. They analyzed the protein content of the water hyacinth, duckweed, and lotus, and found it that it ranged between 8.55% and 14.2%. Adeyemi and Osubor [28] also studied the leaf protein content of water hyacinth and found that it accounted for 50% of its nutrients, showing it to be a suitable material for food.

### 2.2. Hydrothermal Carbonization

The carbon weights obtained for the two plants are shown in Table 2. The weights obtained are very similar. A greater gas concentration was obtained at lower temperatures and a greater quantity of the *Egeria*. Liu and Balasubramanian [31] obtained the lowest yields with HTC (45.3%) with a temperature of 250 °C. Reza et al. [32] concluded that the HTC temperature significantly affects solid hydrochar yield, which decreases as the HTC temperature increases for all feedstocks. The quantity of liquid products is very similar to the water added to the digester for both species, and decreases as the temperature increases.

The calorific value of the carbon products obtained is very similar for both plants (Table 3). Samples treated at higher temperatures have higher calorific values (Table 3). However, the results from the Mann–Whitney test (*L. minor p* > 0.317) and from the Kruskal–Wallis (*E. densa p* > 0.392, *L. minor p* > 0.392) test show no statistical differences between the different temperatures (*p* > 0.05).

These results are very similar to those of Gao et al. [33], who used HTC at 240 °C to obtain hydrochar products from water hyacinth. Their results showed HHVs ranging from 16.83 to 20.63 MJ/kg. Results from the present study are also consistent with those of Zhang et al. (2012) [8], who analyzed the calorific value of duckweed (Araceae) through hydrothermal liquefaction (HTL). Their results showed a calorific value of 34.00 MJ/kg. Although this value is higher, it was achieved at a higher temperature of 340 °C. Other authors have studied the viability of converting biomass into biofuels through HTC. Wang et al. [34] calculated the HHV of *Eucommia ulmoides* and their results ranged from 20.00 MJ/kg at 180 °C to 29.61 MJ/kg at 320 °C. Krylova and Zaitchenko [35] calculated the heating value of plant biomass, such as softwood, hardwood, and pine bark, and found values between 18.50 and 21.00 MJ/kg. Saba et al. [36] also calculated the HHV of *miscanthus* at 230 °C, obtaining a value of 24.60 MJ/kg. Wilk et al. [37] obtained a value for LHV of 20.26 MJ/kg for *Acacia* and 22.64 MJ/kg for *Pine*. These values are higher than those obtained here. Because these values are higher than those obtained from aquatic plants, it could be suggested that the species that were analyzed in this study should be mixed with other sources to increase the heating values.

The ash content is very similar for both species (Table 3). The ash content (Table 3) of the *Egeria densa*-derived hydrochar is higher: It varies with temperature from 33.92% to 49.57%. The ash content of the *Lemna minor* hydrochars is much lower (22.69%–36.51%). To assess the approach, Pearson analysis was used, and the results show a positive correlation (R^2^ ≥ 0.90). A large amount of ash content, as in this study, could cause both environmental and technological problems, such as fine particulate emissions and an increase in volatilization [38]. The carbon products obtained should therefore be mixed with other sources to lower the ash content. This is in concordance with [39], who mixed *Pavlova lutheri*, which has an ash content of 34.5%, with *Miscanthus*, a herbaceous plant.

It is important to highlight that using *L. minor* and *E. densa* to obtain biofuels through HTC is a novel and effective approach that will facilitate the removal of nutrients that cause eutrophication in the waters of the Umia river. Harvesting *E. densa*, which is considered an invasive plant under the Spanish Catalogue of Exotic Invasive Species, should be considered a priority, as it will benefit those species that are affected by its presence.

### 2.3. Palletization

The calorific value of pellets can be seen in Table 4. The *E. densa* sample extracted with acetone showed the highest values, although the samples treated with other solvents have similar results. These values are, however, lower than the values from the carbon products obtained through HTC. This could be because carbon has a greater energy value than the same species when untreated. In the pelletizing process, pellet quality can be affected by various parameters, such as pre-treatment of biomass, moisture content, etc. [40], and consequently their calorific value can also vary.

The Kruskal–Wallis analysis of variance reveals statistically significant differences (*p* < 0.016) when the calorific values of pellets obtained with different solvents (*p* < 0.05) are compared. On the other hand, these results are consistent with those of Munjeri et al. (2015) [41]. They reported the production of briquettes from the aquatic plant *Eichhornia crassipes*, known as the water hyacinth, and compared their results with other briquetted samples, such as eucalyptus, maize, acacia, and pumpkin. Their results are similar to those obtained in the present study, ranging from 14.51 to 20.57 MJ/kg. Miranda et al. (2018) [42] made pellets with microalgae with which they obtained an LHV of 17.98 MJ/kg, higher than the values that were obtained in this study. The results from this study, however, need to be compared with the values established by [43] to check that they meet the relevant requirements (Table 5).

Based on these data, none of the pellets obtained reached the minimum HHV of 14.50 MJ/kg needed for production, so they cannot be used as single feedstock. For a proper comparison, it is also necessary to calculate the ash content and humidity. As with the carbon products obtained through HTC, these pellets could be mixed with other sources to enable the requirements established to be met. Nevertheless, the production of pellets is a low-cost technique that helps reduce biomass waste. However, the main objective was not to obtain financial gain: The idea is not to obtain economic returns from the eutrophication problem. It is important to reduce the contribution of nutrients to the basin to prevent the growth of plants and microalgae that reduce the water quality. To this end, resource managers must take necessary measures, such as controlling use of slurry and fertilizers in areas near the river channel; restoring the riverside forest, which acts as a natural filter; and decreasing the points of contamination (specific and diffuse, etc.). It is necessary to establish a plan for the integral reduction of water pollution.

## 3. Materials and Methods

### 3.1. Sample Preparation

Samples of both *E. densa* and *L. minor* were collected on the banks and surface of the Umia River using water barrels. *E. densa* was collected in Caldas de Reis, and *L. minor* was collected in Ponte Arnelas, both areas in the region of Galicia in north-western Spain.

Samples were washed using a Büchner funnel to eliminate insects, stones, and small branches, and were then placed on a tray with filter paper and oven-dried at 105 °C until completely dry. They were subsequently crushed with a hand blender. A Soxhlet extractor, reproduced from M.D. Luque de Castro & L.E. García Ayuso [44], was used to obtain essential oils. Three solvents were used: Water, ethanol, and acetone (Table 6). Because of the small size of *L. minor* and the technical difficulty of obtaining more biomass, only ethanol was used for this species. All extractions were performed at 60 °C for 60 min. All experiments were performed in triplicate. The product obtained was placed in a rotary evaporator (IKA Rotary Evaporators RV 8 V, Staufen, Germany) to separate the solvent and the solutes by evaporation.

The essential oils obtained as a result of the extraction process were taken to the Centro de Apoyo Científico y Tecnológico a la Investigación (CACTI) to be analyzed. The extraction yield was estimated as follows:

(1) $$Extraction\;yield\;\left(\%\right)=\frac{mass\;of\;total\;extracted}{mass\;of\;dried\;leaves}\;\times \;100\%.$$

The percentages of carbon and nitrogen were determined with a Thermo Scientific Flash EA 1112 (Milan, Italy). A CEM MARSX press was used for the digestion of the samples, in which 0.2 g of the sample was mixed with 1 mL of H_2_O and 10 mL of HNO_3_. A Varian SpectrAA-220 Fast Sequential (Mulgrave, Victoria, Australia) was used to measure the potassium (K) concentration, and a Thermo Scientific XSeries ICP-MS (Massachusetts, USA) was used to measure the concentrations of copper (Cu), nickel (Ni), chromium (Cr), cadmium (Cd), lead (Pb), and selenium (Se). The Kjeldahl method [45] was used to determine the percentage of proteins in the samples, with the nitrogen concentration calculated as a surrogate.

### 3.2. Hydrothermal Carbonization

Hydrothermal carbonization was used to obtain coal from both samples. For each species, different amounts of dried sample and 100 mL of water were weighed and then placed in a digester (Berghof, DAB-3, Tuebingen, Germany). The digester has an inside diameter of 74 mm, an inside height of 183 mm, a volume of 150–210 mL, and can withstand pressure of 200 bar. The digester was placed in a stove for 20 h. For *E. densa*, this was done at four different temperatures: 180, 200, 220, and 240 °C. Due to the small sample size of *L. minor*, HTC was done only at 180 and 240 °C. All experiments were performed in triplicate. The reactor was then placed in water and ice for 20 min to cool. The container was weighed with and without the lid to determine the amount of gas produced. Biochar yields are defined as follows:

(2) $$Biochar\;yield=\frac{Mass\;of\;the\;biochar}{Mass\;of\;the\;raw\;biomass}\;\times \;100\%.$$

A calorimetric bomb (IKA 2000) was used to determine the calorific value of the products obtained according to UNE-EN ISO 18125:2018 [46]. First, the solid residue was placed on a tray and put into a stove at 105 °C for 24 h. The tray was then weighed, the samples were ground, and 1 g was placed in the calorimetric bomb. The calorific value was obtained as in [46].

The higher heating value of the samples examined was determined by using the calorimetric bomb according to UNE-EN 18125:2018 [46], as was the lower heating value, using the results of the elemental analyses of the samples.

To determine the ash content, the method in [47] was followed. For each sample, 1 g was placed in ceramic containers and put into a stove for 1 h at 250 °C and then for 5 h at 550 °C. The ash content was calculated as follows:

(3) $$\%\;ash=\frac{m_{f}}{m_{i}}\cdot 100\%,$$

where *m_i_* is the weight before the samples were introduced in the oven and *m_f_* is the weight after samples were removed from the oven.

### 3.3. Pellet Production

**Figure 1:**
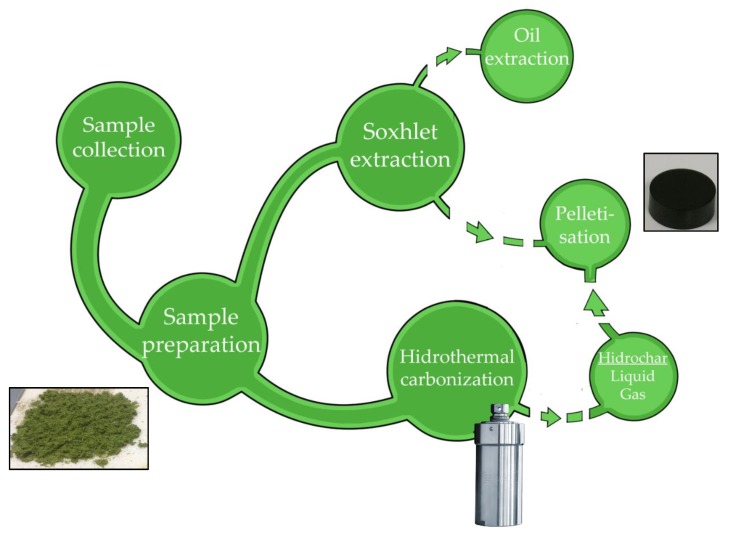
Pellet production process.

A manual pellet press device (EQP-1 Manual Pellet Press, Madrid, Spain) was used to obtain pellets 12 mm in diameter and 15 mm in length (Figure 1), with a mass of 1 g. The sample was then placed in the chamber specially designed for this purpose, the spindle was lowered, and pressure was exerted progressively on the hydraulic lever until the desired pressure of 4 MPa was reached. The volume of the pellets was 1.7 cm^3^ and their density was 588 kg/m^3^. The products used to make the pellets were the carbon obtained from all the products obtained after the Soxhlet extractions and raw samples of *E. densa*. It was not possible to do the same with *L. minor* because there was not enough biomass: It is a very small species, and unfeasibly large field collections would be required to provide the necessary quantity. In the last case, only *E. densa* was used since there was not enough product from *L. minor*. All experiments were performed in triplicate. A calorimetric bomb was used to determine the calorific value of the products obtained.

### 3.4. Statistical Analysis

For the statistical analysis, the Mann–Whitney U-test and Kruskal–Wallis test were used to compare the continuous variables between the groups. This was done using the IBM SPSS Statistics 25 program.

## 4. Conclusions

Carbon products obtained by HTC were obtained and pellets were produced from *E. densa* and *L. minor*. The heating values of the products obtained from HTC were lower (at 14.69 MJ/kg for *Egeria densa* and 17.25 MJ/kg for *Lemna minor* (240 °C)) than those from other sources, such as forest biomass, Moreover, the ash content was found to be high, which could cause boiler problems, but this ash could be used as fertilizer. The heating value of the pellets obtained (with a maximum of 13.49 MJ/kg for *Egeria densa*) failed to meet the requirements set in the relevant standards (14.5 MJ/kg) for use as a single feedstock, so the pellets would need to be mixed with other sources, such as forestry waste. Overall, the results of this novel approach are promising, showing that both species have the potential to be used as biofuels. The use of a soxhlet extractor to obtain essential oils is a simple and effective method. The ability of *L. minor* and *E. densa* to accumulate metals can be considered as a good monitor of river contamination, and harvesting these plants will therefore help to remove excessive nutrients from the ecosystem and thus reduce eutrophication. Finally, to take full advantage of these plants, their protein content could be used as a supplementary food.

## Figures and Tables

**Table 1:** Macro- and microelement concentrations found in extracts of *L. minor* and *E. densa*.

	N	C	K	Cu	Ni	Cr	Cd	Pb	Se
	%	%	g/kg	mg/kg	mg/kg	mg/kg	mg/kg	mg/kg	mg/kg
*Lemna minor*	3.97 ± 0.14	29.63 ± 8.86	23.66 ± 0.35	17.50 ± 0.71	3.50 ± 0.71	5.50 ± 0.71	<1	7.00 ± 0.00	<5
*Egeria densa*	3.665 ± 0.33	29.18 ± 1.96	27.46 ± 0.32	24.00 ± 1.42	9.00 ± 0.00	10.50 ± 0.71	4.00 ± 0.00	12.00 ± 1.41	<5

**Table 2:** Weight of carbon obtained.

Species	T °C	Weight Sample (g)	Hydrochar Obtained (g)	Liquid Obtained (mL)	Gas Produced (g)	Biochar Yield (%)
*Egeria densa*	180 ± 1	10.00 ± 0.01	5.90 ± 0.04	94.21 ± 4.56	0.20 ± 0.05	59.00 ± 0.01
*Egeria densa*	200 ± 1	112.47 ± 0.02	75.23 ± 0.12	93.75 ± 5.60	0.18 ± 0.04	66.89 ± 0.00
*Egeria densa*	220 ± 1	107.7 ± 0.01	65.46 ± 0.21	92.84 ± 2.16	0.18 ± 0.06	60.78 ± 0.00
*Egeria densa*	240 ± 1	102.22 ± 0.01	62.12 ± 0.20	90.21 ± 1.12	0.16 ± 0.02	60.77 ± 0.00
*Lemna minor*	180 ± 1	10.00 ± 0.01	6.14 ± 0.05	94.43 ± 2.43	0.14 ± 0.04	61.40 ± 0.01
*Lemna minor*	240 ± 1	114.02 ± 0.03	73.69 ± 0.14	90.25 ± 3.06	0.12 ± 0.02	64.43 ± 0.00

**Table 3:** Calorific value and ash value (%) of the carbon products obtained through hydrothermal carbonization (HTC). LHV = lower heating value; HHV = higher heating value.

Sample Treatment	LHV (MJ/kg)	HHV (MJ/kg)	Ash Content (%)
*Egeria densa* (180 °C)	12.95 ± 0.73	14.28 ± 1.26	33.92 ± 2.86
*Egeria densa* (200 °C)	14.55 ± 1.12	15.88 ± 1.32	32.30 ± 1.57
*Egeria densa* (220 °C)	13.45 ± 1.36	14.77 ± 0.68	43.67 ± 2.18
*Egeria densa* (240 °C)	13.37 ± 0.97	14.69 ± 0.89	49.57 ± 3.25
*Lemna minor* (180 °C)	15.19 ± 2.11	16.52 ± 1.76	22.69 ± 2.21
*Lemna minor* (240 °C)	15.93 ± 1.68	17.25 ± 1.95	36.51 ± 2.01

**Table 4:** Calorific value of pellets obtained from plants treated with different solvents. LHV = lower heating value; HHV = higher heating value.

Species	Solvent	LHV (MJ/kg)	HHV (MJ/kg)
*Egeria densa*	unmodified	10.75 ± 1.12	12.07 ± 1.35
*Egeria densa*	Ethanol	8.55 ± 0.87	11.38 ± 0.29
*Egeria densa*	Water	10.55 ± 1.36	11.88 ± 1.25
*Egeria densa*	Acetone	12.17 ± 2.29	13.49 ± 2.56
*Egeria densa*	Ethanol	11.71 ± 0.97	13.03 ± 1.33
*Lemna minor*	Ethanol	10.89 ± 1.15	12.21 ± 1.58

**Table 5:** Parameters analyzed in pellet production and regulation value according to the rule established by UNE-EN ISO 17225-6:2014 for solid biofuels.

Parameter	Regulation Value
HHV (MJ/kg)	≥14.5
Ash (%)	≤10
Moisture (%)	<15

**Table 6:** Sample and solvent amounts used to extract essential oils.

Species	Sample Weight (g)	Solvent	Solvent Volume (mL)
*Egeria densa*	2.55	Ethanol	250
*Egeria densa*	2.55	Water	200
*Egeria densa*	10	Acetone	250
*Egeria densa*	5	Ethanol	300
*Lemna minor*	2	Ethanol	200
